# The impact of sacrospinous ligament fixation on pre-existing nocturia and co-existing pelvic floor dysfunction symptoms

**DOI:** 10.1007/s00192-020-04440-z

**Published:** 2020-08-11

**Authors:** Maren Himmler, Aidana Rakhimbayeva, Suzette E. Sutherland, Jan-Paul Roovers, Alexander Yassouridis, Bernhard Liedl

**Affiliations:** 1 Department of Urology, University Medical Center Mannheim, University of Heidelberg, Heidelberg, Germany; 2 Center of Reconstructive Urogenital Surgery, Urologische Klinik Planegg, Planegg, Germany; 3 Department of Urology, UW Medicine Pelvic Health Center, University of Washington, Seattle, WA USA; 4 Academic Medical Center, University of Amsterdam, Amsterdam, The Netherlands; 5 Ethics Committee, Ludwig Maximilian University Munich, Munich, Germany

**Keywords:** Nocturia, Pelvic organ prolapse, Sacrospinous ligament fixation, Overactive bladder, Posterior fornix syndrome, Pelvic floor dysfunction

## Abstract

**Introduction and hypothesis:**

To evaluate whether nocturia and coexisting pelvic floor symptoms in women with pelvic organ prolapse (POP) can be improved by ligamentous fixation of apical vaginal prolapse to the sacrospinous ligament.

**Methods:**

We evaluated the PROPEL study data from 281 women with pelvic organ prolapse stage > 2. Bothersome nocturia and coexisting pelvic floor symptoms were assessed with the Pelvic Floor Disorder Inventory (PFDI) questionnaire preoperatively and at 6, 12 and 24 months after successful vaginal prolapse repair. Women with successful reconstruction (POP-Q stage < 1 at all compartments throughout the 2-year follow-up), defined as anatomical “responders,” were compared to the anatomical “non-responders.”

**Results:**

Among the patients completing all PFDI questions (*N* = 277), anatomical responders and non-responders were the groups of interest for our analysis. We found the occurrence rates of “moderate” or “quite a bit” of nocturia was significantly reduced after surgery in all subgroups (48.7% at baseline vs. 19.5% after 24 months). The occurrence of nocturia was halved for responders compared to non-responders (45.4% and 48.3% at baseline vs. 14% and 29.5% after 24 months). Anatomical non-responders still had a relevant improvement of POP-Q stages, especially in the apical compartment. Prevalence rates of co-existing over- and underactive bladder, fecal incontinence, defecation disorders and pain symptoms were also significantly reduced postoperatively.

**Conclusion:**

Nocturia can be associated with symptomatic POP, with improvements seen following vaginal ligamentous prolapse repair. We caution providers, however, when advising patients of the possible resolution of nocturia following POP reconstruction, that all other traditional etiologies of nocturia must first be ruled out.

## Introduction

Nocturia is a highly prevalent symptom that can lead to loss in quality of life and poor sleep [1, 2]. The incidence is about 2.8% for adults 40–59 years of age and 11.5% for adults > 60 years old [3].

After years of manifold interpretation of definitions, the International Continence Society (ICS) updated the terminology in 2018 and defined nocturia as “The number of times urine is passed during the main sleep period. Having woken to pass urine for the first time, each urination must be followed by sleep or the intention to sleep” [4]. Or, more simply: “The complaint that the individual has to wake at night one or more times to void” [5].

To fully appreciate the possible etiology of nocturia in a woman, a minimum evaluation should include patient history, physical (including pelvic) examination, Pelvic Organ Prolapse Quantification (POP-Q) system measurement and a bladder diary including a frequency-volume-chart (FVC) [6].

Nocturia can occur for many reasons, e.g., 24-h polyuria (24-h urine volume > 40 ml/kg). Nocturnal polyuria (ICS definition: “excessive production of urine during the individual’s main sleep period” [4]) can be a symptom of patients with congestive heart failure, sleep apnea, venous insufficiency, impaired circadian rhythm of vasopressin secretion or excessive evening fluid intake [7–9]. Also, reduced nocturnal bladder capacity, e.g. in patients with overactive bladder (OAB), urinary tract infections, bladder outlet obstruction or cancer, can cause nocturia. The production of urine at night exceeds the nocturnal bladder capacity; therefore, even without excessive urine production, the nocturnal urine volume cannot be stored [8]. Only 32% of patients with nocturia show nocturnal polyuria, so other causes of nocturia must be considered [10]. The symptom nocturia is part of the lower urinary tract symptoms (LUTS) and of the ICS definition of OAB. A recent study by Abu Mahfouz et al. revealed that of their OAB symptoms, 43 of 150 women stated nocturia was the most bothersome of all symptoms [11]. The ICS report mentions POP as a possible clinical etiology for nocturia, and thus it warrants more investigation [9]. Many past studies evaluating nocturia fail to mention or even consider this possible correlation—associated pathophysiology—between POP and LUTS, or POP and nocturia [3, 4, 6, 12].

The possible causal relationship between POP and LUTS/nocturia was first described in 1997 when improvement in these urinary symptoms was noted after successful pelvic floor reconstruction through ligamentous support in 80% of cases evaluated [13]. Therefore, the focus of our analysis was the evaluation of the surgical effect on nocturia and the comparison of the nocturia bother profile before and after surgery by analyzing the data from the PROPEL study.

## Methods

The PROPEL study (ClinicalTrials.gov-Identifier: NCT00638235) was a prospective, observational, multicenter trial, supported by American Medical Systems (AMS), to evaluate the Elevate anterior/apical and Elevate posterior/apical transvaginal surgical mesh devices for POP reconstruction [14]. These devices used macroporous, monofilament polypropylene mesh with attached fixation anchors for standard apical sacrospinous ligament fixation and were inserted all through a single transvaginal incision [15]. All subjects were females, aged > 21 years, with a clinically significant prolapse (symptomatic POP-Q stage > 2) in one or more compartment, anterior, apical and/or posterior. Surgical repairs were performed from May 2006–February 2011. The various exclusion criteria can be seen in the original study publication [14]. Institutional review board approval was granted prior to beginning the original study.

Two hundred seventy-seven of 281 subjects completed all questionnaires and were involved in the analysis with mean age 62.5 (+ 11.6) years in the Elevate posterior/apical group and 63.9 (+ 9.8) years in the Elevate anterior/apical group. The first aim of the PROPEL study was to observe anatomical success, defined as POP-Q stage < 1. The second aim was to assess changes in pelvic floor symptoms following prolapse reconstruction. POP-Q measurements and symptom assessments using the Pelvic Floor Disorder Inventory questionnaires (PFDI) were obtained preoperatively and at 6, 12 and 24 months postoperatively.

The PFDI describes the degree of bother caused by the symptom as “no” = no symptoms, “yes but no bother at all,” “somewhat,” “moderate” and “quite a bit.” Successful anatomical reconstruction was defined utilizing a strict anatomical criterion of POP-Q stage < 1 in all three investigated compartments (apical, anterior and posterior) throughout the 2-year follow-up period; these were termed “responders” (*n* = 141) and were then compared to the “non-responders” (*n* = 87) or all those with POP-Q > 1. Note that the sum of responders and non-responders is not equal to the size of the original sample population. Patients with any missing values in any anatomical region(s) were excluded from the analysis, because they could not be assigned to one or the other group. All statistics were performed using the SPSS program, version 17.0.

We evaluated the surgical effect on nocturia within each of the groups (total population, responder and non-responder) and compared the nocturia bother profile before and after surgery for responders versus non-responders. Additionally, the prevalence rates of coexisting pelvic floor symptoms at baseline and 12 months after surgery were evaluated.

We describe not only the single assessment outcomes, but also the aggregated outcomes including “moderate” and/or “quite a bit” of bother (R2), as these patients have clinically relevant nocturia according to their degree of bother. Statistical analysis was first done to evaluate the significance of a possible effect of surgical POP reconstruction on R2 nocturia subjects as well as other possible differences in the R2 population before and after surgery. Therefore, Cochran’s Q tests were applied in each sample population. In case of significant global effects, McNemar tests were used to localize the simple effects, e.g., the phase pairs with significant differences in the prevalence of R2. The null hypotheses behind the Cochran’s and McNemar tests were (1) prolapse reconstruction does not have a significant long-term effect on the prevalence rates of R2; (2) there is no significant difference between prevalence rates of R2 between phase pairs. Fisher’s exact tests or chi^2^ tests were performed at each time phase when differences in R2 between samples had to be proved as being significant. For comparing equality between two proportions, the approximated (0,1) normal distribution [N (0,1)] was used. Alpha = 0.05 was chosen as the nominal significance level. It was adjusted according to the Bonferroni adjustment whenever a posteriori tests (like the tests on simple effects) had to be done.

## Results

### Pre- and postoperative distribution of the POP-Q-stages within the different anatomical regions

Of those completing PFDI questions, 277 were suitable for analysis. The patient baseline demographics can be seen in the original study data (mean age, race, BMI, gravidity, parity, prior hysterectomy, menopausal status, vaginal estrogen usage prior to surgery) [16].

Table 1 shows the relative frequencies of the various POP-Q stages (0–4) in the different anatomical regions (anterior, apical, posterior) before and after reconstruction for the responder and non-responder populations, as well as the total population, respectively. There was no significant difference in the baseline POP-Q scores between the responder and non-responder groups except for two cases, marked with * (*p* < 0.05, chi^2^-tests). According to the definition, responders were those with POP-Q stage < 1 in any compartment at any time; non-responders were those with POP-Q stages > 1. In contrast to the stable anatomical results of the responders, the non-responders had POP recurrences (POP-Q stages > 2) in the anterior region in 41.8% (at 6 months), 45.1% (at 12 months) and 66.1% (at 24 months) and in the posterior region in 29% (at 6 months), 30.5% (at 12 months) and 27.1% (at 24 months). Recurrences in the apical region were rare: 4.8% (at 6 months), 6.1% (at 12 months) and 6.8% (at 12 months). When considering the total population, we see recurrence of POP-Q stages ≥ 2 in the anterior, apical and posterior anatomical regions of maximally 28%, 6% and 13%, respectively.

**Table 1:**
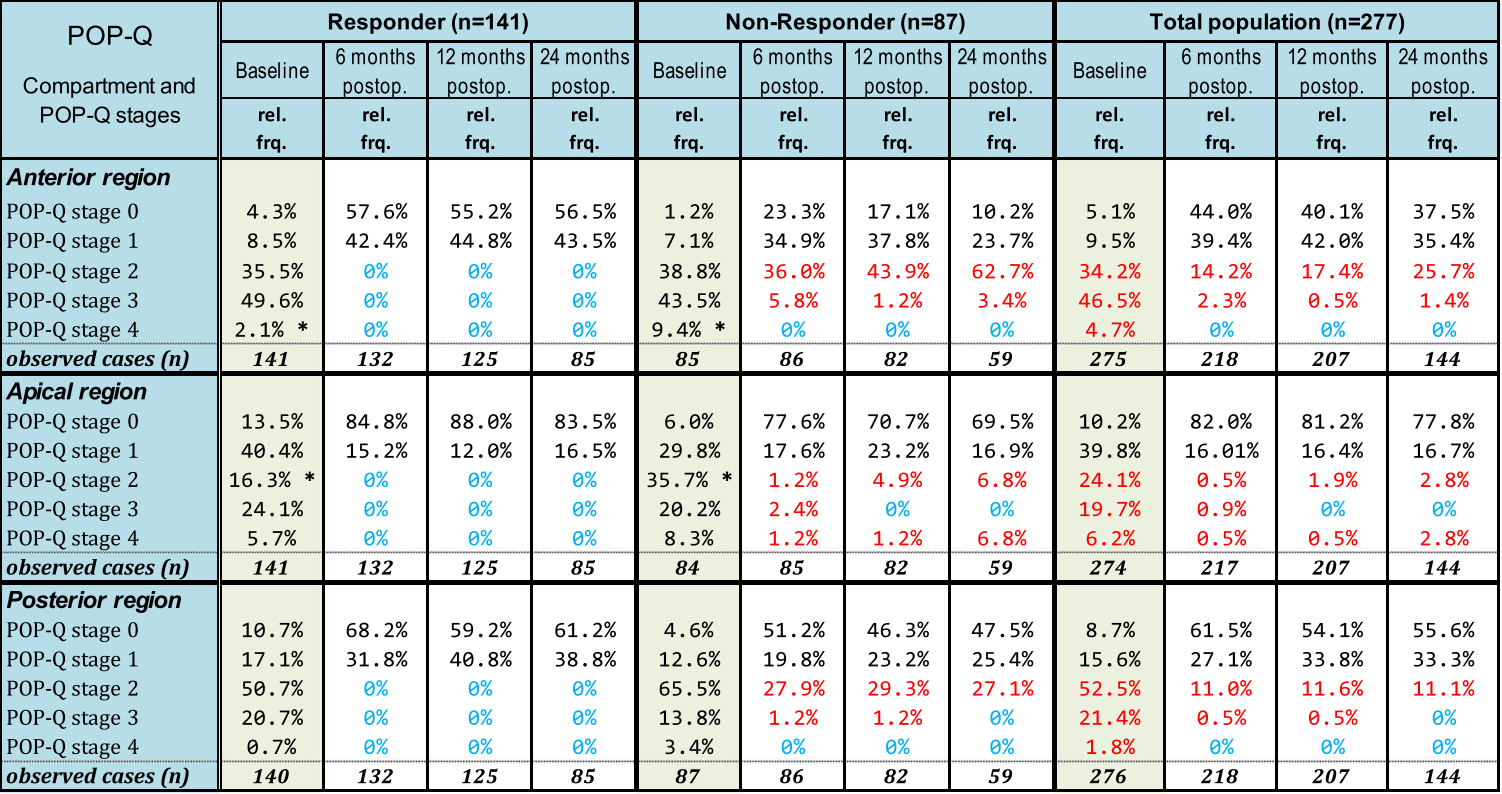
Relative frequencies (rel. Frq.) of the POP-Q stages before (baseline) and 6, 12 and 24 months postoperatively (postop.) for the responders (*n* = 141), non-responders (*n* = 87) and total population (*n* = 277) after differentiation into the anatomical compartments: anterior, apical and posterior. Total numbers (*n*) of observed cases are shown. In red and blue, relative frequencies of the occurrence and non-occurrence rates of POP-Q stages > 2 at baseline and follow-up. By comparing the relative frequencies for each single stage with chi^2^-tests or the approximated N (0,1)-normal distribution, we did not find significant differences in almost all (except for two, marked with *) cases. Therefore, the responders did not differ significantly from the non-responders regarding the preoperative POP-Q stage distribution

### Nocturia and its pre- and postoperative prevalence rates

**Fig. 1:**
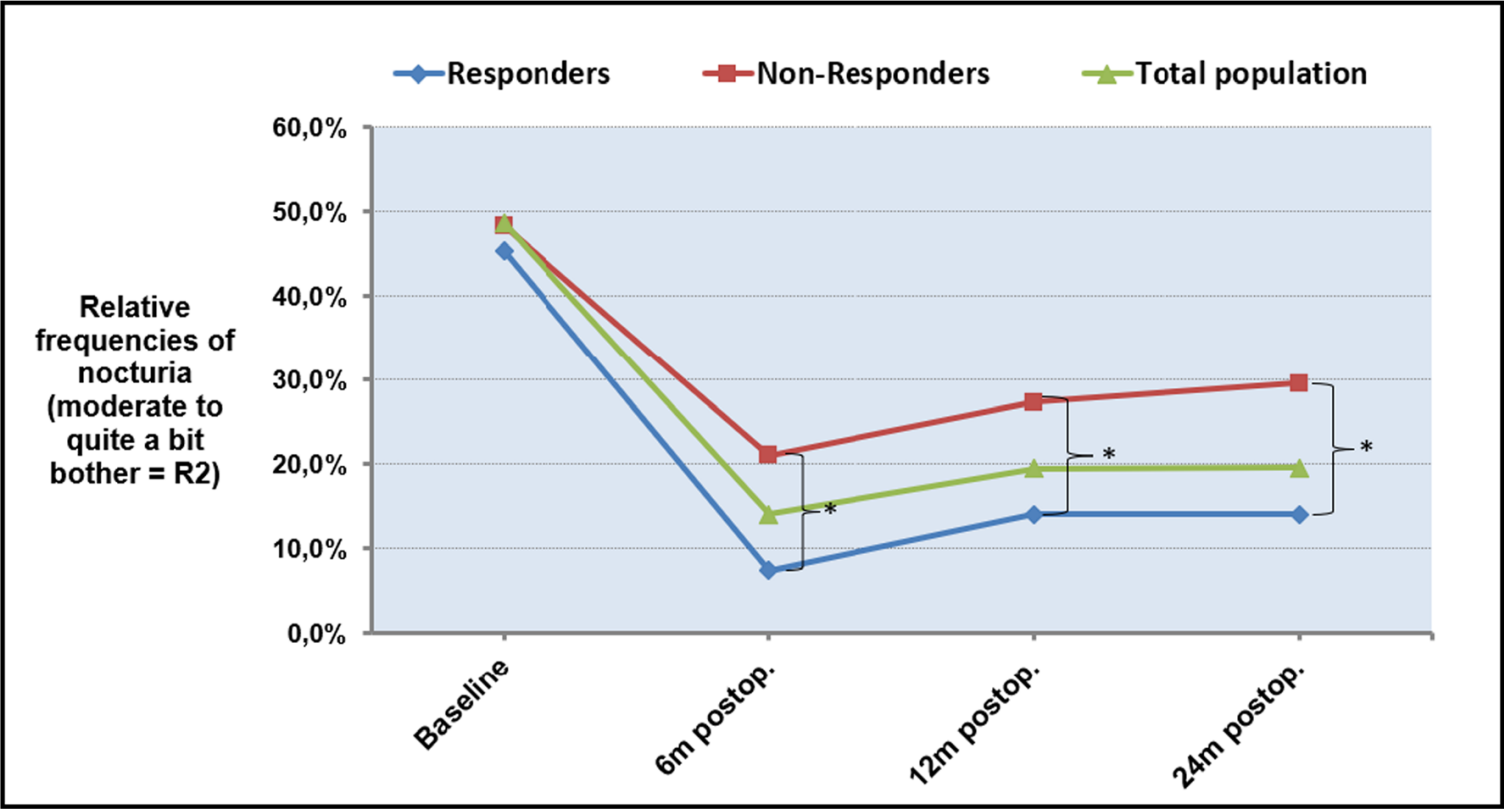
Courses of the relative frequency of R2 from baseline to 6, 12 and 24 months after POP reconstruction for responders, non-responders and the total population. The square brackets with * indicate statistically significant differences between the responder and non-responder samples (chi^2^-tests, *p* < 0.05). m = months; postop. = postoperatively

Table 2 and Fig. 1 show the prevalence of the single and composed outcome R2 (nocturia of “moderate” and/or “quite a bit” of bother) at baseline and the different postoperative phases regarding the original population, responders and non-responders. At baseline, frequencies of R2 were similar in all groups: 48.7% vs. 45.4% vs. 48.3% for the total population vs. responders vs. non-responders, meaning almost half of the patients were suffering from moderate or quite a bit nocturia. The occurrence rates of R2 were reduced significantly after surgery in all sample populations [Cochran’s Q tests, *p* values < α*, where α* is a Bonferroni corrected α (= 0.05)]. Twenty-four months after surgery, frequencies of R2 decreased to about 20% for the total population, 14% for the responders and about 30% for the non-responders. Analysis of the simple effects revealed that the differences in the occurrence rates of R2 were significant between each postoperative phase and baseline, but not between any pairs of the postoperative phases [McNemar tests, *p* values < α*, where α* is a Bonferroni-corrected α (= 0.05)].

**Table 2:**
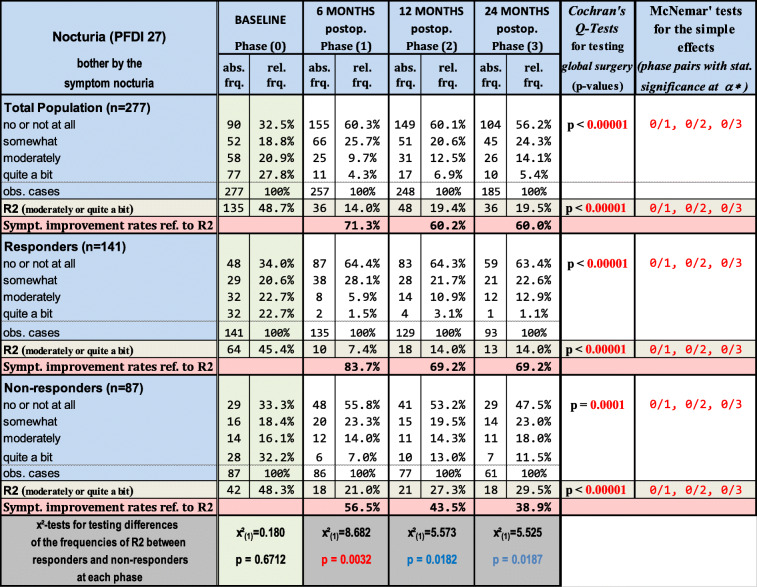
Absolute (abs. Frq.) and relative frequencies (rel. Frq., in %) of the outcomes for the bother from nocturia as “no or not at all,” “somewhat,” “moderate” and “quite a bit” and of the composed outcome R2 (“moderate” or “quite a bit”) in the total population (*n* = 277), the responders (*n* = 141) and the non-responders (*n* = 87) as well. Additionally, the symptom-free rates referred (ref.) to R2 at 6, 12 and 24 months after surgery are listed. Blue- and red-colored *p* values or number pairs indicate statistical significances at the nominal level of significance of 0.05 (at a Bonferroni-corrected level of significance α*, where α* < a = 0.05). obs. = observed

Additionally, the symptom-free rates of R2 over time are shown. The results of inferential statistical analysis comparing responders with non-responders regarding the prevalence rates of R2 are presented as well. Performing the chi^2^-tests, significant or marginally significant differences between responders and non-responders were found at each phase. The improvements seen in the R2 group after surgery were significantly higher in the responders (83.7% at 6 months, 69.2% at 24 months) than in the non-responders (56.5% at 6 months, 38.9% at 24 months).

### Nocturia and coexisting pelvic floor symptoms

We were interested in understanding to what extent women with POP and nocturia also had other pelvic floor symptoms and the subsequent effect of POP reconstruction on those symptoms. Therefore, we examined the simultaneous occurrence of some coexisting “moderate” or “quite a bit” of pelvic floor symptoms (= R2_c_) in women with “moderate” or “quite a bit” of nocturia (= R2_n_) at baseline and 12 months after surgery (Table 3). Symptom frequencies of R2_c_ for over- and underactive bladder as well as fecal incontinence, defecation disorders and pain at baseline and 12 months after surgery are shown. Also, the percentages of symptom-free rates are presented. Note that of the women showing R2_n_ at baseline (N_B_ = 135), 16 patients were lost to follow-up 12 months after surgery (N_12m_ = 119).

**Table 3:**
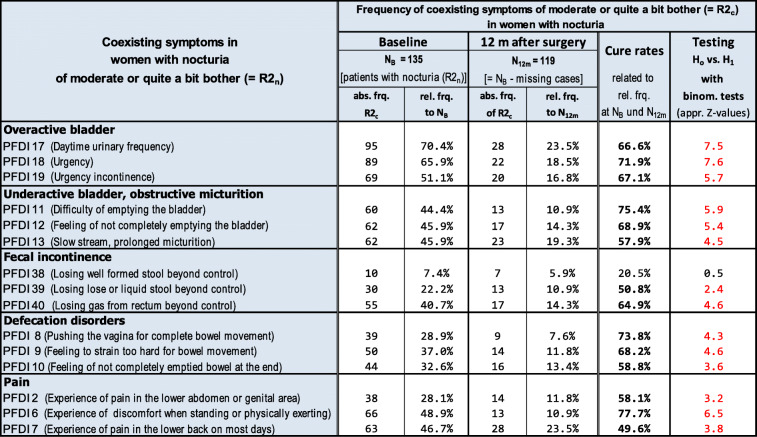
Frequencies of various coexisting symptoms of “moderate” or “quite a bit” of bother (R2_c_) in women with nocturia of “moderate” or “quite a bit” of bother (R2_n_) at baseline and 12 months after surgery. Also, symptom-free rates 12 months after surgery are shown. Z-values in red [> than the critical z-value (2.290) at α = 0.01] indicate significant reduction of the frequency of R2_c_ 12 months after surgery compared to baseline. N_B_ = number of patients at baseline; N_12m_ = number of patients 12 months after surgery; appr. = approximated; abs. = absolute; rel. = relative; frq. = frequencies

For each coexisting symptom, the z-values of the approximated normal distribution were calculated when testing the hypothesis H_0_: P (rel. Frq. of R to N_B_) ≤ P (rel. Frq. of R to N_12m_) vs. H_1_: P (rel. Frq. of R to N_B_) > P (rel. Frq. of R to N_12m_). All red-colored z-values of the considered coexisting symptoms were higher than the critical z-value of α = 0.01, except for one z-value of PFDI 38 (z_(0.01)_ = 2.290). Therefore, the hypothesis H_0_ can be rejected in favor of H_1_, indicating a significant postoperative reduction of R2_c_ for all coexisting symptoms 12 months after surgery, except PFDI 38 (i.e., PFDI 17 daytime urinary frequency 70.4% to 23.5%, PFDI 18 urgency 65.9% to 18.4%).

**Fig. 2:**
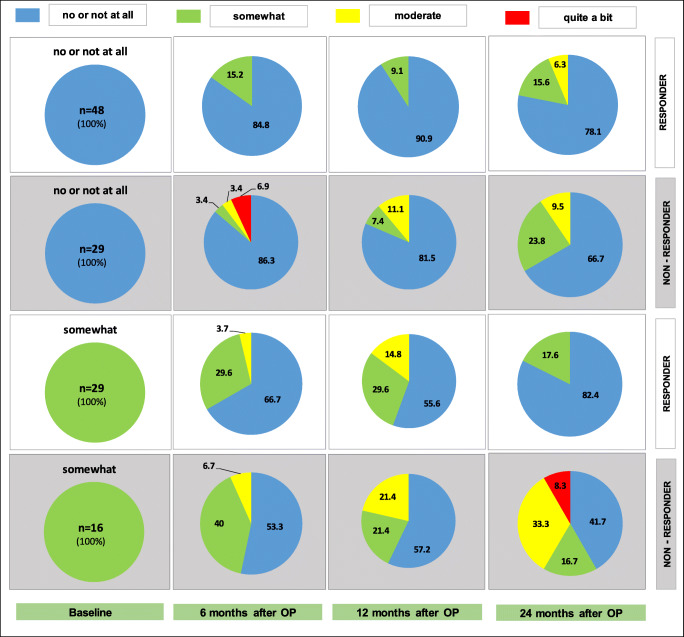
Change of bother severities 6, 12 and 24 months (in %) after POP reconstruction in the population of responders and non-responders for patients with baseline nocturia bother of “no,” “not at all” or “somewhat.” *n* = total number; postop. = postoperatively

**Fig. 3:**
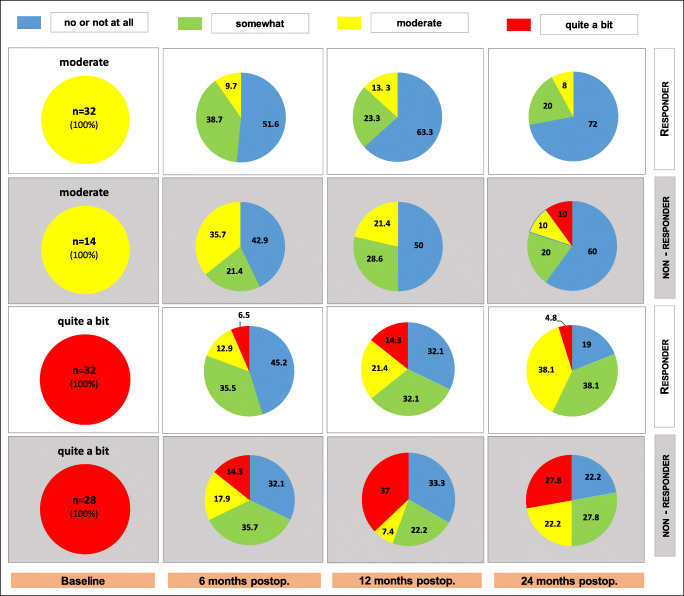
Change of bother severities 6, 12 and 24 months (in %) after POP reconstruction in the population of responders and non-responders for patients noting baseline nocturia bother of “moderate” or “quite a bit.” *n* = total number; postop. = postoperatively

In Figs. 2 and 3, we show the changes in bother severity for nocturia (PFDI-27) 6, 12 and 24 months after surgery for responders and non-responders.

Figure 2 shows that 78.1% of the responders with baseline PFDI-27 bother of “none” or “not at all” remained so 24 months after surgery; 15.6% and 6.3% of these patients reported “somewhat” or “moderate,” and none of them reported “quite a bit.” Of the responders with baseline PFDI-27 bother of “somewhat,” 82.4% improved to “no” or “not at all,” 17.6% remained at “somewhat,” and none worsened to “moderate” or “quite a bit” by 24 months postoperatively.

Figure 3 shows that of the responders with baseline PFDI-27 of “moderate” bother, 72.0% improved to “no” or “not at all,” 20.0% to “somewhat,” and 8.0% remained at “moderate,” with none worsening to “quite a bit” out to 24 months. Thus, 92.0% of the patients experienced an improvement in their nocturia symptoms following POP reconstruction out to 24 months after surgery.

Of the responders with baseline PFDI-27 bother of “quite a bit,” 19.0% improved to “no” or “not at all,” 38.1% to “somewhat,” 38.1% to “moderate,” and only 4.8% experienced no change and remained at “quite a bit” at 24 months after surgery. Thus, 57.1% of the patients with “quite a bit” of nocturia bother experienced considerable improvement out to 24 months following POP repair.

Of the non-responders (postoperative POP-Q > 1) with baseline PFDI-27 bother of “moderate,” 60.0% improved to “none” or “not at all,” 20.0% to “somewhat,” 10.0% remained at “moderate,” and 10.0% worsened to “quite a bit” 24 months after surgery. Thus, 80.0% of these anatomical non-responder patients with “moderate” bother nocturia preoperatively noted an improvement in these symptoms at 24 months postoperatively.

## Discussion

Nocturia, urgency, pelvic pain, abnormal bladder emptying and other pelvic floor-related symptoms can be caused by POP [17]. These symptoms often co-exist and can be summarized as “posterior fornix syndrome” (PFS) [18, 19]. Defects of the posterior zone can cause OAB, which includes the symptom nocturia [20]. The pathophysiology of symptom development has previously been summarized by Petros and Liedl [19, 21]. Ligamentous and vaginal laxities cause POP and dislodgement of the muscle insertion points. As striated muscles are composed of sarcomeres, overstretching them causes rapid reduction of muscle force and therefore muscular dysfunction [22]. Urgency and nocturia can occur when vaginal laxities result in reduced muscle force. Distention of the bladder base activates stretch receptors at smaller than usual bladder volumes causing an inappropriate bladder contraction—this is interpreted as “urgency” [19]. Elevate anterior/apical and Elevate posterior/apical provide stellar apical support through strong fixation at the sacrospinous ligament with very successful anatomical results (Table 1).

When counseling women about their nocturia, it is very important to evaluate the exact symptom complex they are presenting and also the bother severity [6]. Depending on the findings, performing urodynamics and using specific questionnaires might give additional information that help counseling these patients [2]. That way, all other possible etiologies of nocturia should be ruled out before POP can be determined to be its cause. These investigations are the key to proper patient selection, which results in optimal surgical results and symptom cure rates.

A recent study of Hagovska et al. showed that a 3-month physical exercising program in overweight young women with OAB symptoms could significantly reduce nocturia in the treatment group compared to the control group [23]. Prior studies have shown a similar effect, even though the anatomical parameters of POP did not change [24]. Therefore, obese women should be counseled to do physical exercising before considering surgery. If possible, conservative measures should always be chosen over surgery.

Understanding that connective tissue laxities and defects can be the link between the pathophysiology of POP and the cause of coexisting symptoms, it then stands to reason that ligamentous repair of POP may improve or even cure many of these symptoms in some women [17]. Accordingly, Liedl and Goeschen noted improvements of up to 95% in all POP-related symptoms by providing a good anatomical mesh-supported reconstruction with fixation at the sacrospinous, sacrouterine and/or cardinal ligaments [25].

In our analysis, bothersome coexisting symptoms were significantly more frequent in women with nocturia bother of “moderate” or “quite a bit” (R2_n_) compared to women with minor or no complaints. The connective tissue laxities may be responsible for this difference.

Sivaslioglu et al. reported improvements in a small population of 30 women who were treated with a posterior sling (infracoccygeal sacropexy) for POP repair. Coexisting symptoms including nocturia were evaluated pre- and postoperatively. Cure rates of 86% were reported for nocturia, 82% for pelvic pain, 75% for urgency and 93% for obstructed micturition after surgery [26].

Richardson reviewed the facts about surgical cure of nocturia in the past years and found that suspension of the vaginal apex, whether posterior sling or sacrocolpopexy, leads to an improvement of nocturia and associated posterior zone symptoms in 60.5%–86% [17].

Liedl et al. also reported cure rates between 60% and 80% for major complaints of nocturia, which were consistent out to 24 months. Additionally, patients with anatomical defects in the posterior zone (apex) who later received Elevate-posterior/apical had significantly more nocturia preoperatively than those with a defect in the anterior zone alone (cystocele). They found similar complaints of nocturia in patients with POP-Q stage 2 vs. POP-Q stage 3–4 [20].

In 2004, Goeschen et al. published prospective data of 83 women with POP and PFS, who were treated with an infracoccygeal sling procedure (posterior sling). They found cure rates of 78% for nocturia. In 2015, Goeschen changed the technique of fixation and repeated the study with 198 patients (modified posterior sling). The cure rates for nocturia (81%) remained essentially the same [18]. Therefore, one can hypothesize that the method of fixation is not as important as the successful fixation itself. A recent study of de Castro et al. affirms these findings comparing two different surgical approaches (vaginal vs. abdominal mesh supported apical fixation) that were similarly efficacious in objective and subjective cure rates [27].

Caliskan et al. in 2018 reported data of 368 women with POP, who were treated with the same two operative methods just mentioned. Cure rates for nocturia were 63% (modified posterior sling) vs. 62% (original posterior sling) [28].

Natale et al. treated 272 patients with high levator myorrhaphy to cure the apical defect. Most of these patients (247) also received a mesh to repair the cystocele. They showed nocturia rates of 45% at baseline, which decreased significantly to 34% after surgery [29].

The significantly higher symptom-free rates in responders with optimal anatomical reconstruction in all compartments (POP-Q < 1) compared to non-responders indicate that the high rates of cystoceles (up to 60%) and rectoceles (about 24%) of mainly stage 2 could be responsible for the lower improvement rates in the non-responder group of this study.

In this study, the main aim was to assess the anatomical success (defined very strictly as POP-Q < 1) and the potential complications associated with POP repair utilizing transvaginal mesh [16, 30]. Quality of life and symptom improvement, which is at least as important as anatomical repair, was only set as a secondary outcome measure. Another limitation was that 49 patients of the original population had to be excluded because of missing data and could therefore not be fairly assigned to a group.

This study shows that correct anatomical reconstruction with mesh-supported ligamentous fixation significantly reduced nocturia and other pelvic floor symptoms due to POP during a 2-year follow-up after surgery. These data and all other papers cited here that report improvements after POP repair are gained from women willing to undergo surgery for their symptomatic POP. Although this concept would benefit from further study, women should be counseled about the high probability of nocturia resolution, along with other pelvic floor symptoms, following reconstructions of their symptomatic POP, especially when their POP involves the posterior compartment (apex, posterior wall and/or perineal body). Exclusion and treatment of other possible etiologies of nocturia are certainly recommended before considering corrective POP surgery directed at also improving the nocturia symptoms.

## Conclusions

Patients with advanced pelvic organ prolapse are frequently bothered by nocturia, a relationship that is not well addressed in the current literature. We show that ligamentous vaginal prolapse repair can, apart from correcting the anatomy, reduce the bother related to associated pelvic floor symptoms, including nocturia. We caution providers, however, when counseling patients about the possible resolution of nocturia following POP reconstruction, that all other traditional etiologies of nocturia must first be ruled out. Proper patient selection is the key.
